# Development of 3D Printing Filament from Poly(Lactic Acid) and Cassava Pulp Composite with Epoxy Compatibilizer

**DOI:** 10.3390/polym17233228

**Published:** 2025-12-04

**Authors:** Thidarat Kanthiya, Pattraporn Changsuwan, Krittameth Kiattipornpithak, Pornchai Rachtanapun, Sarinthip Thanakkasaranee, Pensak Jantrawut, Nuttapol Tanadchangsaeng, Patnarin Worajittiphon, Thorsak Kittikorn, Kittisak Jantanasakulwong

**Affiliations:** 1 Office of Research Administration, Chiang Mai University, Chiang Mai 50200, Thailand; thidaratkanthiya05@gmail.com (T.K.); first200294@gmail.com (K.K.); 2 Faculty of Agro-Industry, Chiang Mai University, Mae-Hea, Mueang, Chiang Mai 50100, Thailand; pornchai.r@cmu.ac.th (P.R.); sarinthip.t@cmu.ac.th (S.T.); 3 Center of Excellence in Materials Science and Technology, Chiang Mai University, Chiang Mai 50200, Thailand; 4 Center of Excellence in Agro Bio-Circular-Green Industry (Agro BCG), Chiang Mai University, Chiang Mai 50100, Thailand; 5 Faculty of Pharmacy, Chiang Mai University, Chiang Mai 50200, Thailand; pensak.amuamu@gmail.com; 6 College of Biomedical Engineering, Rangsit University, Pathumthani 12000, Thailand; nuttapol.t@rsu.ac.th; 7 Department of Chemistry, Faculty of Science, Chiang Mai University, Chiang Mai 50200, Thailand; patnarin.w@cmu.ac.th; 8 Department of Materials Science and Technology, Faculty of Science, Prince of Songkla University, Songkhla 90110, Thailand; thorsak.k@psu.ac.th

**Keywords:** waste of cassava, 3D printing, polymer composite, fiber, poly(lactic acid), agricultural biodiversity

## Abstract

A 3D printing filament was fabricated from poly(lactic acid) (PLA), cassava pulp (CP), and epoxy using a twin-screw extruder. Several bio-composites were synthesized by varying the amount of epoxy (0.5, 1.0, 3.0, 5.0, and 10.0 wt.%). The size of the CP fibers significantly affected the surface quality, filament diameter, and mechanical properties of the final product. The smallest fiber size (45 µm) provided a smooth surface and consistent diameter. Incorporating 1 wt.% of epoxy into PLA/CP enhanced the tensile strength (56.6 MPa), elongation at break (6.2%), and hydrophobicity of the composite. The composite mechanical properties deteriorated at epoxy contents above 1 wt.% due to the amplified plasticizer effect of excessive epoxy. The optimized PLA/CP/epoxy formulation was used to generate the 3D filament. The resultant filament displayed a tensile strength of 64.6 MPa and elongation at break of 9.8%, attributed to the fine morphology achieved via thorough mixing provided by the twin-screw extruder. Epoxide-mediated crosslinking between PLA and CP enabled the development of a novel 3D printing filament with excellent mechanical properties. This research illustrates how agricultural residues can be upcycled into high-performance biomaterials with innovation in sustainable manufacturing, inclusive economic growth, reducing reliance on petroleum-based plastics and thus providing benefits regarding human health, climate change mitigation, plastic in the ocean, and environmental impacts.

## 1. Introduction

Thermoplastics are highly versatile materials widely applied in numerous industries owing to their unique properties, such as high strength-to-weight ratios, ease of processing, and their ability to be remolded after melting. Thermoplastics are excellent gas- and water-barrier materials, making them ideal for packaging foods and beverages to prevent oxidation and spoilage. However, a dramatic increase in thermoplastic consumption has raised environmental concerns. Replacing petroleum-based plastics with those made from renewable resources is crucial for mitigating environmental impact [1]. Bioplastics are natural polymers derived from organic materials, some of which are biodegradable. They are categorized as first, second, or third generation depending on whether the renewable material is derived from a food or non-food source [2]. Polylactic acid (PLA) is a biodegradable and bioactive thermoplastic polyester derived from renewable sources such as corn starch, cassava, and sugar cane [3,4,5]. PLA exhibits inherent biodegradability, as its ester backbone can be hydrolyzed and subsequently broken down by microorganisms under suitable composting conditions, especially in environments with elevated temperature and humidity [6]. Although PLA is the most widely used raw material in extrusion-based three-dimensional (3D) printing owing to its environmental friendliness, it has several drawbacks, including high cost, lower strength and durability, poor heat resistance, and limited flexibility [7,8]. Additive manufacturing (AM), also known as 3D printing, constructs 3D objects through layer-by-layer material deposition to form the desired shape. This technique enables the direct creation of complex structures from digital models, which cannot be achieved using conventional manufacturing methods, such as milling, molding, coating, and forming. Three-dimensional printing techniques do not require any physical changes in the tool or machine after designing the product using software, and reduce material waste relative to subtractive manufacturing approaches. The wide spectrum of materials that have been created using 3D printing include plastic components, nonfunctional human organs, and superconducting materials. Three-dimensional printed polymer products are rarely used for structural and functional applications because of their inadequate mechanical properties and inherent electrical and thermal insulating characteristics. The addition of reinforcement materials such as nanoparticles and short or continuous fibers has been widely investigated to overcome the mechanical performance limitations of 3D printed polymers [9].

Cassava pulp (CP) is an important and inexpensive agro-industrial solid byproduct of cassava starch production. In recent years, the industrial production of cassava starch in Thailand has increased significantly (current production, 1.5–2.2 million tons per year), and the price of cassava pulp is 250–550 THB/ton [10,11]. CP contains a high proportion of holocellulose (~66%) and lignocellulosic fiber (~14–50 wt.%), giving it a cellulose structure comparable to sugarcane bagasse and bamboo fibers. CP provides effective reinforcement in polymer composites and can achieve strength improvements similar to these fillers. Its finer and more uniform fiber morphology allows better dispersion and stress transfer, which often results in more efficient strengthening than bagasse or bamboo. CP also requires less preprocessing, making it a practical and competitive natural fiber filler with strong mechanical reinforcement capability. However, the critical limitation of using natural fibers as reinforcements is the lack of compatibility between the fibers and matrix. When subjected to external stresses, composite materials undergo various types of degradation, leading to deteriorated mechanical performance. Therefore, modifying the surfaces of natural fibers plays a crucial role in improving their adherence to the matrix and reducing moisture absorption [12]. Several techniques, such as alkaline, silane, and chemical treatments, have been applied to increase the compatibility between the fiber and polymer matrix. Furthermore, polymer composite materials that combine favorable properties such as excellent deformation and strength characteristics with low specific weight are widely applied in manufacturing [13]. Epoxy oligomers, which act as plasticizers, are the most commonly used binders in industry. They are commonly used to manufacture adhesives and coatings [14]. The plasticizing effect stems from the introduction of epoxidized double bonds into the polymer structure, resulting in improved material properties. In addition to their plasticizer role, epoxy compounds are also used as synthetic crosslinkers, particularly for crosslinking proteins, owing to the reactivity of epoxide functional groups with amino, carboxyl, and hydroxyl groups [15]. However, the use of epoxy resins for the reactive blending of PLA and CP to prepare 3D filaments has not been investigated. Moreover, optimizing the properties of PLA/CP composites through reactivity control has not been comprehensively elucidated.

This study aimed to design and fabricate biocomposite materials for 3D printing by combining PLA, CP, and epoxy resin via reactive melt-blending. This innovative approach enabled the fabrication of 3D filaments with excellent mechanical properties and toughness, delivering a wood texture. Furthermore, the effects of the fiber grain size and epoxy resin content on the morphology and mechanical properties of the PLA/CP composites were investigated. Short CP fibers were selected for toughening PLA, and their mechanical properties, reactivity, water contact angle, morphology, thermal properties, and folding were observed. The optimum reactant ratio was selected to fabricate 3D filaments using a twin-screw extruder, and their mechanical and thermal properties were compared with those of a compression-molded sample. This study presents, for the first time, a PLA/CP biocomposite filament reinforced through epoxy-mediated reactive crosslinking, enabling significantly improved toughness and printability compared with conventional PLA-based materials. The integration of agricultural waste fibers with controlled epoxy reactivity offers a novel pathway for producing sustainable, high-performance 3D-printing filaments using a simple melt-blending process. The PLA/CP/epoxy filament developed in this work enables advanced applications in lightweight structural parts, precision engineering prototypes, and customized biomedical devices. Improved interfacial adhesion and tunable toughness achieved through epoxy-mediated crosslinking further support its use in high-performance additive manufacturing. These enhancements position the material as a promising sustainable alternative for next-generation functional components.

## 2. Materials and Methods

### 2.1. Materials

Polylactic acid 4032D with MW 100,000 g/mol, a density of 1.24 g/cm^3^, and MFI 7 g/10 min at 210 °C was purchased from PTT global chemical Public Co., Ltd., Bangkok, Thailand. High Mw (100,000 g/mol) with low MFI (7 g/10 min at 210 °C) was selected due to excellent processing ability for 3D filament fabrication. Diglycidyl ether of bisphenol A grade 0302 of epoxy resin was purchased from EASY Resin Co., Ltd., Nonthaburi, Thailand. CP was obtained from Premier Quality Starch Co., Ltd., Mukdhahan, Thailand. The α-amylase enzyme was purchased from Reach Biotechnology Co., Ltd., Pathum Thani, Thailand. Iodine solution (purity 1%, AR grade) was purchased from S.Y. Trading Ltd., partnership, Surat Thani, Thailand. Analytical reagent-grade deionized water and sodium hydroxide (NaOH) were purchased from Chem House Ltd., Bangkok, Thailand.

### 2.2. Preparation of Cassava Pulp for Starch Removal

CP was washed and dried before removing the starch. Deionized water (100 mL) was added to 100 g of CP. All components were mixed before adjusting the pH of the mixture to 6.5–7.0, followed by the addition of 20 IU/mL of α-amylase enzyme. The mixture was stirred and heated at 90 °C for 20 min. The treated CP was tested for the presence of starch using an iodine solution.

### 2.3. Alkali Treatment of Cassava Pulp

CP was prepared without bleaching to reduce chemical treatments. The CP was dried using a hot-air oven for 48 h at 80 °C, pre-treated with NaOH 12 wt.% aqueous solution at 1:20 pulp ratio, and boiled for 3 h at 85 °C to remove fatty acid, hemicellulose, residual lignin, and other impurities. Fiber surface treatment was performed to prepare the pure fiber surface to react with epoxy and PLA matrix. Low degradation fiber surface was obtained by alkali treatment without bleaching or strong acid treatment. The samples were dissolved in distilled water and dried in a hot-air oven. The dried samples were sieved to obtain particles smaller than 250 microns.

### 2.4. Sample Preparation

The PLA sample was melt-blended with diglycidyl ether of bisphenol A of epoxy resin using two-roll mills (Squeeze 6520 Precision resource Co., Ltd., Bangkok, Thailand) at a speed of 50 rpm and temperature of 160 °C for 5 min. CP and epoxy were mixed for 15 min. To ensure uniform mixing, PLA, CP, and epoxy were blended under high shear using a two-roll mill at controlled temperature and rotation speed. The shear conditions were sufficient to disperse epoxy uniformly even at low concentrations (0.5–5.0 wt.%). The homogeneity of mixing was further confirmed by the consistent mechanical properties and the absence of unmixed epoxy domains in the SEM micrographs. The sheet samples were compressed using hot compression at 160 °C for 10 min, followed by quenching in cool water.

### 2.5. Tensile Properties

Tensile strength and elongation at break were observed using tensile testing machine (MCT–1150; A&D Company Limited, Tokyo, Japan). The dog-bone test was performed follow JIS K 6251–7 standard [16]. The sample were 5 mm in width, 30 mm in length, and 1 mm in thickness. The tensile tests were performed at a strain rate of 10 mm/min to ensure consistent deformation behavior across all samples. The samples were kept at 50 RH and 25 °C for 24 h. The results for each sample were averaged over five experimental runs.

### 2.6. Fourier-Transform Infrared Spectroscopy (FTIR)

The ATR method based on Fourier transform infrared spectroscopy (FTIR/IR–4700, Jasco Corp., Tokyo, Japan) was used to study the reaction mechanism. The samples were prepared as a thin film with a thickness of 100 µm. The IR spectra were obtained from 4000 to 500 cm^−1^ with a resolution of 4 cm^−1^.

### 2.7. Differential Scanning Calorimetry (DSC)

Thermal analyses were investigated using a differential calorimeter (DSC 823E; Mettler Toledo, OH, USA). Samples (5–10 mg) were placed in a closed aluminum pan. DSC analysis was performed from 0 to 200 °C with a heating and cooling rate at 10 °C min^−1^.

### 2.8. Contact Angle

The contact angles were measured using a DSA30E Krüss GmbH instrument, Hamburg, Germany. Water contact angle was observed using drop shape analysis. Water was dropped onto the film surface, and images were recorded every 20 s for 10 min.

### 2.9. Scanning Electron Microscopy (SEM)

A scanning electron microscope (SEM, JEOL JSM– 5910LV JEOL Co., Ltd., Tokyo, Japan) was used to examine the morphology at a magnification of 2000× and an accelerating voltage of 15 KV. The samples were broken in liquid nitrogen and coated with a thin layer of gold before testing.

### 2.10. Melt Flow Index (MFI)

The melt flow index (MFI) of PLA, PLA/CP, and PLA/CP/epoxy composites was measured using a standard melt flow indexer at 210 °C under a 2.16 kg load, following the ASTM D1238 procedure [17]. Each sample was preheated for 5 min prior to measurement to ensure thermal equilibration, and the flow rate was recorded in g/10 min. These conditions allow direct comparison of melt viscosity among different formulations and enable assessment of the competing effects of crosslinking and plasticization induced by epoxy at various concentrations.

### 2.11. Folding Endurance

Folding cycles of samples were studied using a Gotech GT 6014 A (Gotech Testing Machines, Inc., Taichung, Taiwan). The dimensions of samples were 100 mm × 15 × 1 mm^3^. The grip clearance was 10 mm. All samples were observed by averaging five specimens for each sample.

### 2.12. Statistical Analysis

One-way analysis of variance (ANOVA) was investigated using the Statistical Package for Social Sciences (SPSS Version 17; Armonk, NY, USA). Duncan’s test was used to evaluated the differences (*p* < 0.05).

## 3. Results

### 3.1. FTIR Analysis of Fiber

**Figure 1:**
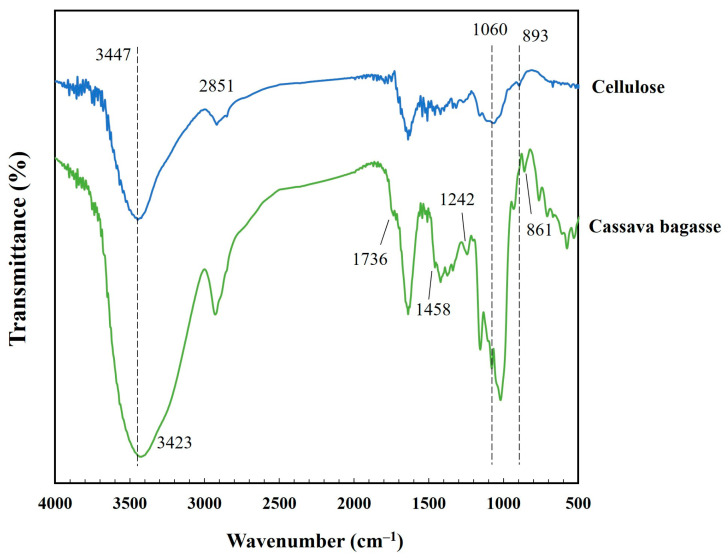
FTIR spectra of cassava pulp (green line) and cellulose (blue line).

FTIR was used to identify cellulose functional groups in natural CP and chemically modified cassava fibers, as shown in Figure 1. The lignocellulosic materials (green line) show characteristic transmittance peaks at 1736, 1458, 1242, and 758 cm^−1^, corresponding to C=O stretching, C=C aromatic skeleton vibration, C–O–C stretching and C–H aromatic, respectively [18,19]. The C–O–C vibrations on 700–900 cm^−1^ region associated with carbohydrate on fiber structure [20]. The peak at approximately 3423 cm^−1^ was assigned to OH stretching [21]. However, some of the above peaks were not detected after chemical modification of CP. The peak at 861 of cassava bagasse assigned to C–O–C of the remained starch, which was removed after chemical and enzyme treatments. The cellulose fiber (blue color) spectrum displayed bands at 3347, 2851, 1060, and 893 cm^−1^ arising from O–H stretching, C–H stretching, C–O–C anti symmetric, and C–H, respectively [22,23]. The lower peak intensities in the cellulose spectra compared to those of the CP are a consequence of lignin removal during the alkaline treatment process.

### 3.2. Effect of Fiber Size on the Mechanical Properties of PLA/CP Composites

#### 3.2.1. Mechanical Properties of PLA/CP Composites

**Figure 2:**
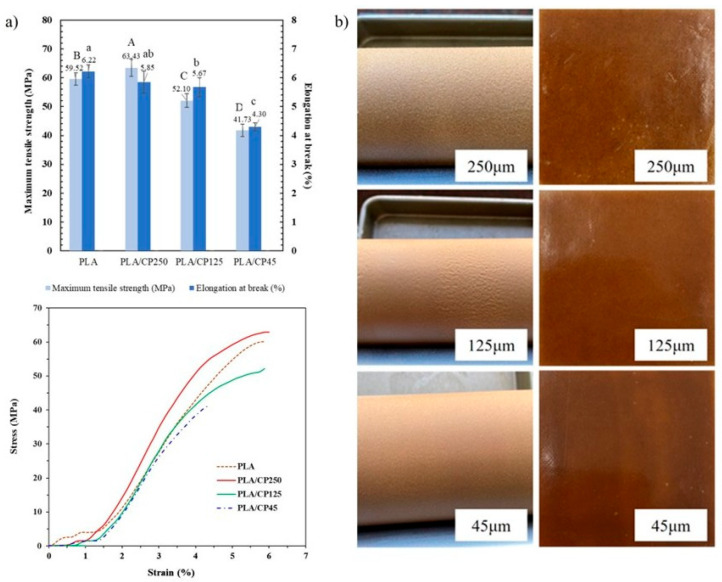
Properties of PLA/CP composites fabricated using 45, 125, and 250 µm-length CP fibers: (**a**) tensile properties (**b**) images of samples showing surface smoothness. Different uppercase letters denote statistically significant differences in maximum tensile strength, while different lowercase letters indicate significant differences in elongation at break (*p* < 0.05).

PLA composites (polymer/fiber ratio, 90:10 *w*/*w*) were fabricated using CP fibers of varying length (45–250 µm) employing two roll mill machines. The effects of fiber length on the mechanical properties of the PLA composites are shown in Figure 2a. The maximum tensile strength and elongation at break decreased with decreasing fiber size. Thus, the highest maximum tensile strength (63.4 MPa) was attained by the PLA composite with 250 µm CP fiber length. The PLA composites with fiber lengths of 125 and 45 µm exhibited tensile strengths of 52.1 and 41.7 MPa, respectively. Interfacial adhesion between the polymer and fiber surface decreases with decreasing fiber length, leading to inferior tensile properties [24]. On the other hand, optimal mixing was observed for the 45 µm fiber, delivering the smoothest surface among the three polymer composites (Figure 2b). The fine distribution of fibers and superior surface smoothness may allow for greater consistency of the 3D filament diameter. Optimizing the mechanical properties of a composite with a smooth surface is crucial for developing 3D printer filaments. Although the 45 µm CP fibers did not provide the highest mechanical strength, they offered the most uniform dispersion during melt blending, which greatly improved processability for filament extrusion. The finer fiber size facilitated smoother flow and produced filaments with more consistent diameter and surface quality compared with longer fibers. Therefore, the 45 µm fiber was deemed optimal as it provided high tensile strength and the smoothest surface.

#### 3.2.2. Morphology of PLA/CP Composite

**Figure 3:**
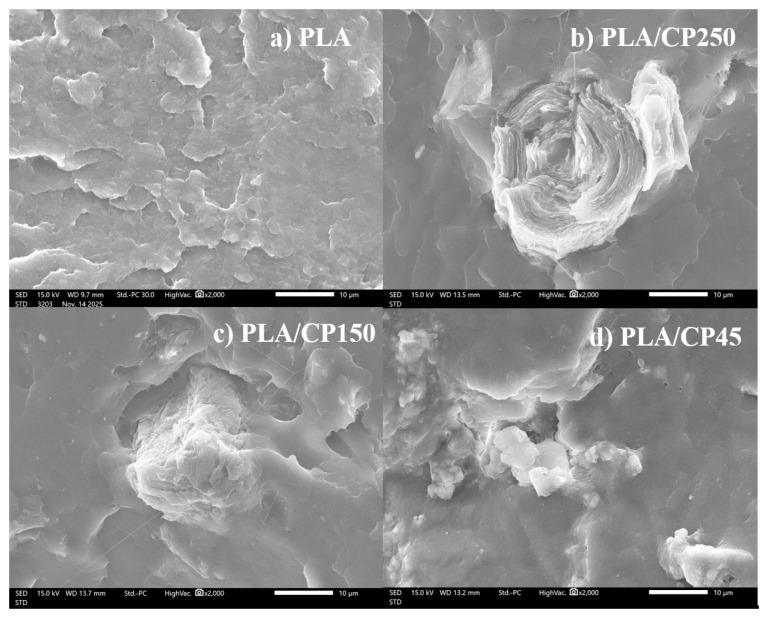
SEM images of PLA/CP composites comprising (**a**) PLA, (**b**) 250 µm, (**c**) 125 µm, and (**d**) 45 µm CP fibers.

The SEM cross-section images of the fabricated PLA/CP composites are shown in Figure 3. The material properties of the PLA/CP matrices differed from those of pure PLA and were affected by the CP fiber length. The 250 µm CP fiber exhibited poor bonding with the PLA matrix (Figure 3a). Furthermore, although the longer fiber length improved the stiffness of the composite this led to increased brittleness. Reducing the CP fiber length improved the smoothness of the PLA/CP matrix surface. The shortest fibers (45 µm, Figure 3c) acted as particles and formed agglomerative clusters in the PLA/CP matrix, thereby mitigating the low compatibility between PLA and CP fibers.

### 3.3. Effect of Epoxy Resin Content on PLA/CP/Epoxy Resin Composite Properties

#### 3.3.1. FTIR Analysis of PLA/CP/Epoxy Composite

**Figure 4:**
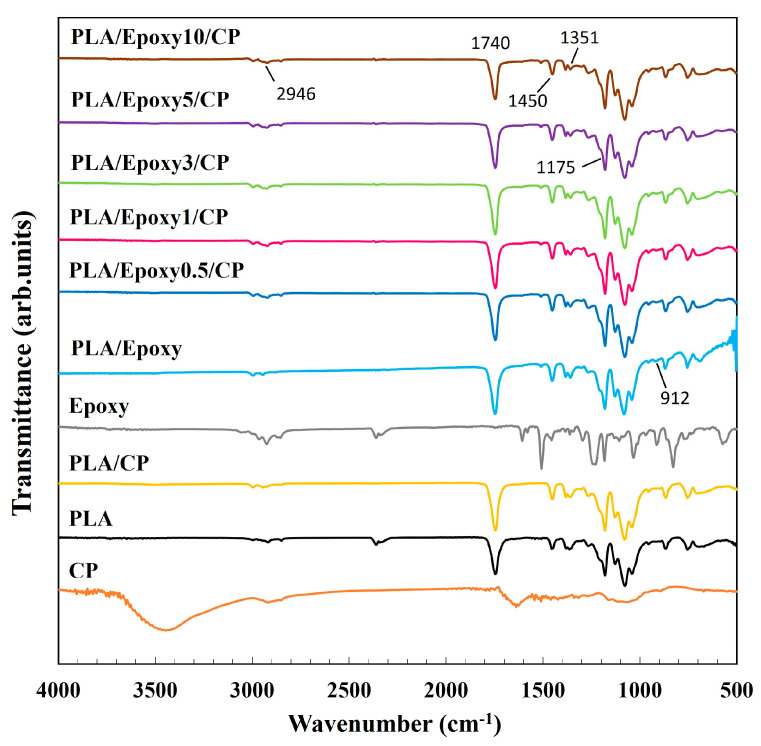
FTIR spectra of PLA/CP composites containing varying amounts of epoxy.

**Figure 5:**
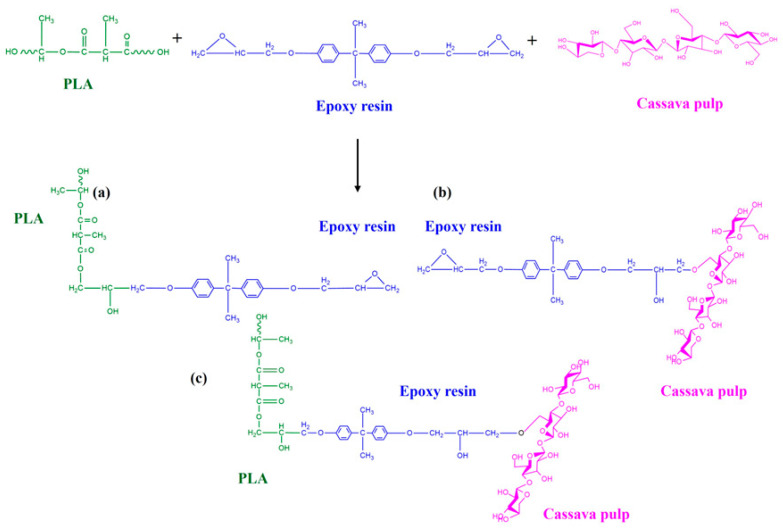
Scheme illustration of potential interactions among PLA, epoxy resin, and cassava pulp (**a**) Proposed reaction between PLA and epoxy resin (**b**) Proposed reaction between epoxy and cassava pulp (**c**) Proposed ternary reaction involving PLA, epoxy resin, and cassava pulp.

The functional group changes resulting from the incorporation of CP and epoxy into the PLA matrix were investigated using FTIR analysis. Figure 4 shows the IR spectra of pure PLA, pure CP, PLA/epoxy, and PLA/CP composites containing 0.5, 1.0, 3.0, 5.0, and 10.0 wt.% epoxy. Pure PLA gave rise to peaks at 2946, 1740, 1450, and 1175 cm^−1^, corresponding to C–H stretching, C=O stretching, –CH_3_ bending, and C-C stretching vibrations, respectively (Table 1). Characteristics O-H, C–H, and C–O–C stretching bands of CP appeared at 3447, 2821, and 1060 and 893 cm^−1^, respectively [22,23]. The peak at 912 cm^−1^ in the epoxy resin spectrum corresponded to asymmetric C–O stretching of the oxirane ring, while the aromatic C=C stretching bands were observed at 1510 and 1609 cm^−1^ [25]; the aromatic C–H vibration of bisphenol A appeared at 3059 cm^−1^ [25]. The PLA/CP composite spectrum displayed C–H, C=O stretching, C–O–C absorption, and –OH stretching bands at 1450–1378, 1740, 1086–1175, and 3500–4000 cm^−1^, respectively [26]. The PLA/epoxy sample exhibited aromatic C=C and asymmetric C–O oxirane stretching absorptions at 1510 and 912 cm^−1^, respectively. In the PLA/CP/epoxy sample, the intensity of the characteristic oxirane peak at 912 cm^−1^ was lower than that in PLA/epoxy because the epoxide groups can react with the hydroxyl groups present in the CP fiber [27]. The interaction between –OH of CP and epoxy ring opening was also indicated. The epoxy groups reacted with the –COOH of PLA and the –OH groups of CP to connect PLA and CP, which enhanced the mechanical properties of the composite. A schematic of expected reaction between PLA and CP via epoxy is presented in Figure 5.

#### 3.3.2. Mechanical Properties of PLA/CP/Epoxy Resin Composite

**Figure 6:**
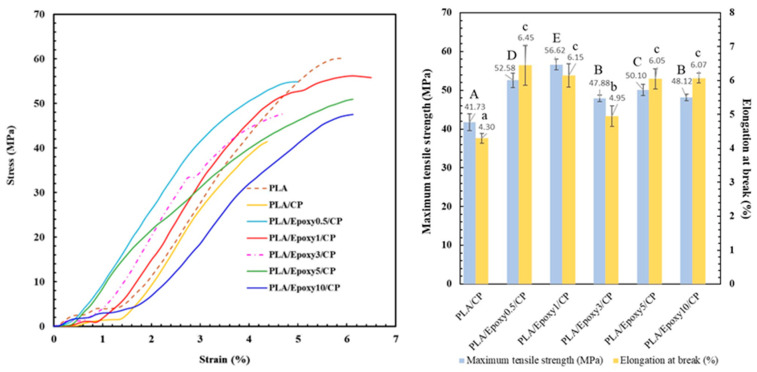
Tensile strengths and elongation at break of PLA/CP composites containing 0.5, 1.0, 3.0, and 5.0 wt.% epoxy. Different uppercase letters denote statistically significant differences in maximum tensile strength, while different lowercase letters indicate significant differences in elongation at break (*p* < 0.05).

The effects of epoxy content (0.5, 1, 3, 5, and 10 wt.%) on the mechanical properties of the composite materials was investigated. The materials were generated by the sequential mixing of PLA, epoxy, and CP fiber. Figure 6 shows the effects of epoxy addition on the tensile strength and elongation at the break of the PLA/CP composites. The addition of epoxy to PLA/CP led to improved tensile strength and elongation at break due to efficient crosslinking between PLA and the epoxy polymer, while CP acted as reinforcement for the composite material [28,29,30]. The 1 wt.% epoxy sample exhibited the highest tensile strength (56.6 MPa) and elongation at break (6.2%), implying optimal crosslinking. Epoxy contents exceeding 1 wt.% resulted in inferior tensile properties due to excessive plasticizer effects [31]. Based on these results, the combination of PLA, CP with 45 µm fiber length, and 1 wt.% epoxy was chosen to prepare the 3D printer filament.

#### 3.3.3. Contact Angle of PLA/CP/Epoxy Composite

**Figure 7:**
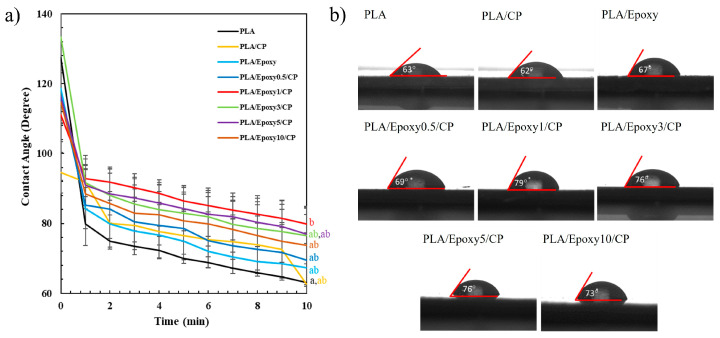
Plot of water contact angles of (**a**) PLA composites with varying epoxy contents during 0–10 min contact time; (**b**) contact angles of PLA composites with varying epoxy contents at 10 min contact time. Lowercase letters indicate significant differences of contact angle at 10 min (*p* < 0.05).

The effects of epoxy addition on the hydrophilicity of the composite materials was investigated. Figure 7a illustrates contact angles of PLA, PLA/CP, and PLA/CP with 0.5–10 wt.% epoxy measured over 10 min. The contact angles of PLA, PLA/CP, PLA/epoxy, and PLA/CP/epoxy 0.5, 1, 3, 5, and 10 wt.% at 10 min were 63°, 62°, 67°, 69°, 79°, 76°, 76°, and 73°, respectively (Figure 7b). The contact angles of all samples decreased with contact duration. PLA exhibited the lowest contact angle. PLA is sensitive to moisture which provides low water contact angle 50–80° by its surface tension [32]. The roughness of the small CP fibers in PLA/CP and crosslinking in PLA/epoxy resulted in greater contact angles. The PLA/CP composite incorporating 1 wt.% epoxy displayed the highest contact angle as a result of optimum crosslinking between PLA and CP enabled by the epoxy group. The contact angles of the PLA/CP composites comprising 3–10 wt.% epoxy were lower because of the hydrophilic nature of the epoxy group [33]. Optimal crosslinking occurred at 1 wt.% epoxy as the available epoxide groups fully reacted with the limited –COOH and –OH groups of PLA and CP, respectively, whereas higher epoxy contents left excess unreacted epoxide acting primarily as a plasticizer. The decrease in contact angle at >1 wt.% epoxy was attributed to these unreacted polar groups increasing the surface energy rather than to phase separation.

#### 3.3.4. Morphology

**Figure 8:**
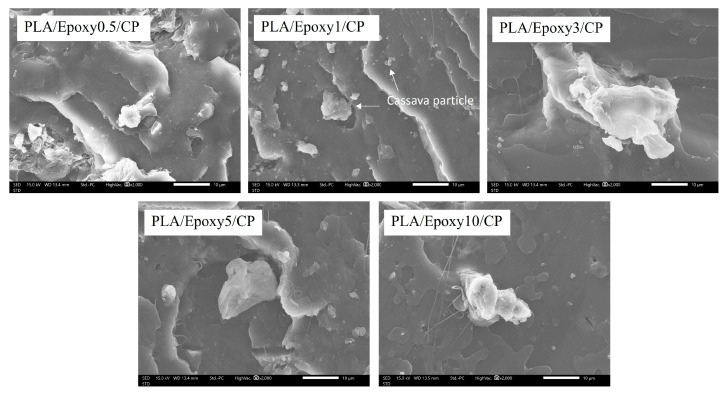
SEM images of PLA/CP/Epoxy composites with varying epoxy contents (0.5–10 wt.%).

The mechanical, flexural, and thermal properties of the PLA/CP/Epoxy composites are significantly affected by CP dispersion characteristics. SEM was used to evaluate the cracking surfaces of the PLA/CP/epoxy samples and the CP distribution therein (Figure 8). PLA/CP/Epoxy0.5 exhibited a rough cracking surface and partial CP fiber removal A fine dispersion of small CP fibers was observed in the PLA/Epoxy1/CP sample. This indicates that 1 wt.% epoxy enabled optimal crosslinking between PLA and CP, resulting in well-dispersed small-sized fibers in the PLA matrix. The PLA/Epoxy3-10/CP samples displayed large CP aggregates, resulting from increased inter-fiber connectivity enabled by excessive epoxy. The clusters of fibers acted as defects and reduced the tensile strength. Overall, the addition of 1 wt.% epoxy as a crosslinking agent delivered a robust composite surface and improved the tensile strength [34]. The excellent mechanical properties and CP distribution in PLA/Epoxy1/CP make it suitable for fabricating 3D printing filaments.

#### 3.3.5. Melt Flow Index

**Figure 9:**
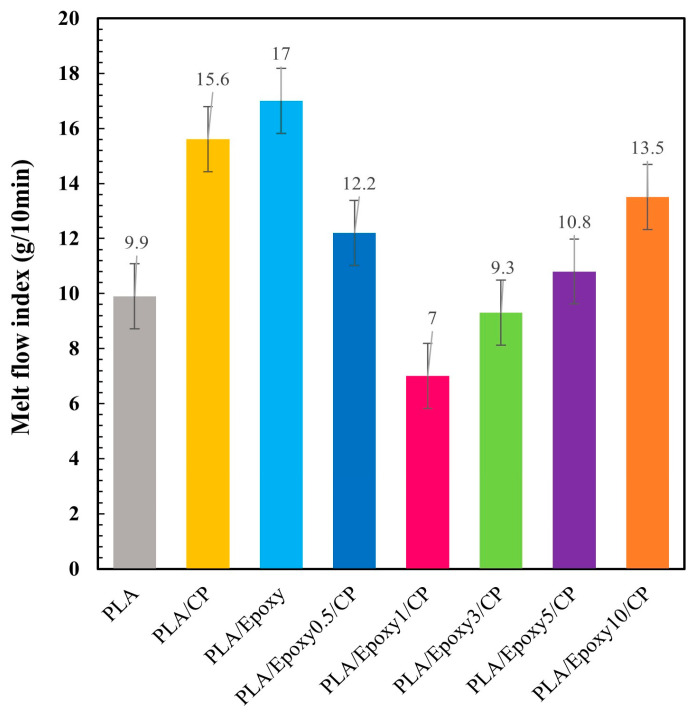
Melt flow index (MFI) values of PLA/Epoxy/CP composites with varying epoxy contents (wt.%).

The melt flow index (MFI) is used to assess the flowability or melt viscosity of a polymer. It provides valuable information for estimating the behavior of a material during the forming processes. The effects of epoxy addition on the MFI of the bio-composite materials was evaluated using the MFI test, as shown in Figure 9. The MFI value of the pure PLA was 10 g/10 min, whereas that of the PLA/CP sample increased to 15.8 g/10 min. The higher MFI value of the composite material stemmed from the combined effects of the low thermal resistance of PLA, high disorientation of the fiber, and incompatibility between the PLA matrix and CP. The MFI of PLA90/Epoxy10 was also high because at that concentration, the epoxy additive behaved more like a plasticizer than a crosslinking agent. The PLA/Epoxy0.5/CP and PLA/Epoxy1/CP composites showed lower MFIs due to the effects of crosslinking, whereas the plasticizing effect of excessive epoxy (3–10 wt.%) increased the MFI. Optimizing the amount of epoxy is essential for maximizing the crosslinking of PLA and minimizing its plasticizer effects, which impart flexibility and flowability to PLA [35].

#### 3.3.6. Folding Endurance Test

**Figure 10:**
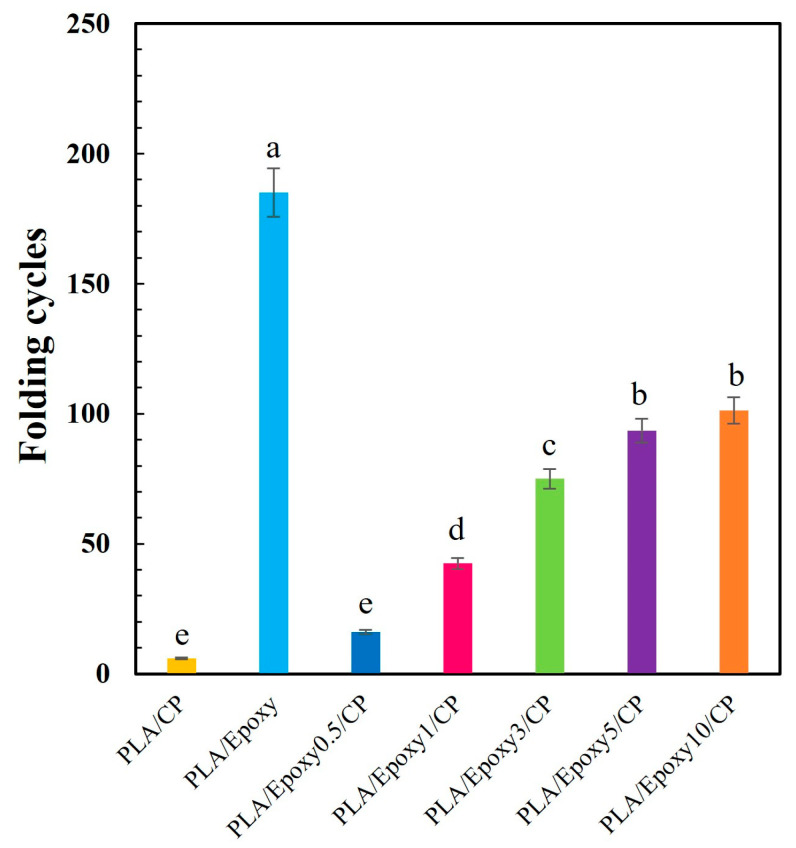
Folding cycles of PLA/CP, PLA/epoxy, and PLA/epoxy/CP composites with 0.5–10 wt.% epoxy. Significantly different mean values (*p* < 0.05) are indicated by distinct lowercase letters.

The folding endurance (FE) test has been suggested as a suitable method for evaluating the actual strength of composite films. PLA/CP endured only five folding cycles due to the brittleness of PLA and the incompatibility of PLA/CP, whereas PLA/Epoxy remained intact after 185 cycles owing to the crosslinking and plasticizing effects of the epoxy additive. The folding resistance of the PLA/Epoxy/CP samples increased with increasing epoxy content; specifically, composites containing 0.5, 1.0, 3.0, 5.0, and 10.0 wt.% epoxy underwent 16, 42, 75, 94 and 101 folding cycles, respectively (Figure 10). The epoxide groups of the epoxy resin reacted with the carboxylic groups of the PLA matrix and the hydroxyl groups of the CP, thereby linking the two components. Crosslinking within the PLA matrix and between PLA and CP improved the toughness and folding resistance of the PLA/Epoxy/CP samples. The epoxy resin acted as both a crosslinking agent and a plasticizer for PLA, which improved its tensile strength, elongation at break, and toughness [36]. The high folding resistance and excellent tensile properties of PLA/Epoxy/CP composites are beneficial for reducing the brittleness of 3D filaments. The brittle 3D filaments are broken during fabrication process while high toughness 3D filaments provide better processing efficiency. Therefore, optimal tensile strength with high toughness is considered for 3D filament preparation.

#### 3.3.7. Thermal Properties

**Figure 11:**
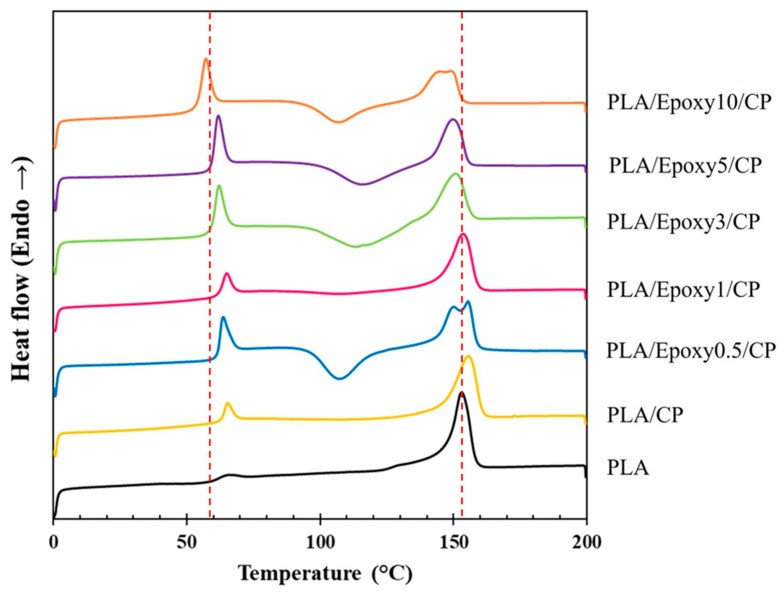
Second-scan DSC thermograms of PLA, PLA/CP, and PLA/Epoxy/CP composites.

The thermal properties of the composites were evaluated using differential scanning calorimetry (DSC). These measurements were used to monitor the individual phase transitions of PLA and PLA/Epoxy/CP composites, as shown in Figure 11. From the thermograph obtained for the second heating of pure PLA, the glass transition temperature (T_g_) and melting temperature (T_m_) were 63.3 and 153.3 °C, respectively. The T_g_ and T_m_ values of PLA/CP were similar to those of pure PLA. The T_g_ values of PLA/CP containing 0.5, 1, 3, 5, and 10 wt.% epoxy showed a decreasing trend (61.2, 61.1, 59.2, 58.6 and 53.7 °C, respectively). Epoxy acts as a plasticizer for PLA, imparting softness and flexibility, and increases the free volume between the polymer chains [36]. The thermal characteristics of the PLA, PLA/CP, and PLA/Epoxy/CP composites are shown in Table 2. The T_m_ and T_g_ values decreased with increasing epoxy content. PLA and PLA/CP displayed high degrees of crystallinity (36.1% and 30.7%, respectively) owing to melt-crystallization during the cooling process after the 1st scan, but did not show recrystallization peaks. In the PLA/Epoxy/CP composites, the chain length of the polymer was extended through epoxide-mediated crosslinking, and PLA chain mobility was suppressed. This phenomenon reduces PLA crystallization. However, PLA/Epoxy1/CP showed a high crystallinity of 28% because the elongated PLA chain underwent high-melt crystallization during cooling after the 1st scan without recrystallization during the 2nd scan. At higher epoxy contents (3–10%), plasticizer effects were dominant, which prevented melt-crystal formation during cooling after 1st scan. The decreasing T_g_ of PLA/Epoxy/CP with increasing epoxy content confirms the plasticizing effect of the epoxy resin in the PLA polymer. The decrease in crystallinity at higher epoxy contents was attributed to the plasticizing effect, which increased chain mobility and disrupted orderly chain packing during cooling. This was supported by the reduction in ΔHm and ΔHc values from the DSC analysis, confirming that excess epoxy hinders crystal formation while simultaneously lowering Tg through enhanced chain flexibility. CP and epoxy distinctly influenced the crystallization behavior of PLA. CP provided lignocellulosic surfaces that act as heterogeneous nucleating sites, which promoted cold crystallization. The presence of fiber reduced chain mobility and alters crystallinity depending on its dispersion. The addition of epoxy further restricted PLA chain movement through chain extension or branching effects, shifting the cold-crystallization peak and generally lowering crystallinity. CP and epoxy modified both thermal transitions and crystalline structure of PLA by combining nucleation effects with reduced chain mobility. The combined effects of crosslinking and plasticizer functionality improved the thermal properties of the PLA/epoxy/CP composites.

#### 3.3.8. Three-Dimensional Filament Derived from PLA/Epoxy1/CP

**Figure 12:**
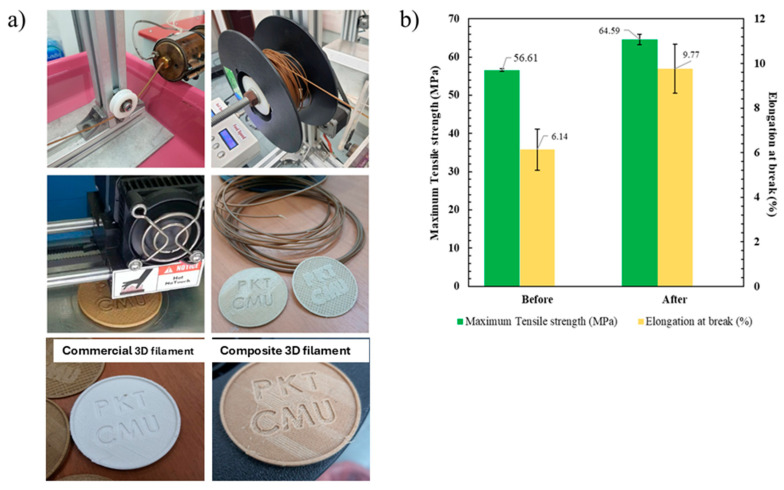
(**a**) 3D filament generation using a twin screw extruder and (**b**) Tensile strength and elongation at break of PLA/Epoxy1/CP composites generated via twin-screw extrusion and hot compression.

The PLA/Epoxy1/CP formulation exhibited optimal mechanical, thermal, and toughness properties, and was thus selected to fabricate a 3D filament using a twin-screw extruder (Figure 12a). Figure 12b shows the tensile properties of the samples obtained via hot compression and 3D filament fabrication. The tensile strength of the 3D filament (64.6 MPa) was higher that of the hot compression specimen (56.6 MPa). The 3D filament also showed improved elongation at break compared to that of the normal specimen (9.8% and 6.1%, respectively). The enhanced tensile properties of the 3D filament sample fabricated via twin-screw extrusion compared to that via two-roll mill mixing originated from the fine mixing by the high-shear-rate twin-screw extruder. Scaling up for 3D filament composite was effectively prepared using twin-screw extruder. A 3D filament with favorable tensile properties and high toughness was obtained by controlling the high-shear mixing process, the reaction of epoxy in the mixture, and the plasticizing effect of epoxy on the PLA/CP composites. The sample plate was printed using PLA/CP composite filament to confirm processing ability of composite filament (Figure 12a).

## 4. Conclusions

A 3D filament based on PLA/Epoxy/CP composite was successfully developed, wherein epoxy resin acted as a plasticizer and crosslinking agent, and cassava pulp (CP) acted as a reinforcement material. Chemically treated cassava fibers of varying lengths (45–250 µm) were employed, revealing that 45 µm-length fibers provided the smoothest surface and imparted excellent melt processing ability and mechanical properties to the PLA/CP composite, which are beneficial for controlling the 3D filament diameter in the printing machine. FTIR analyses confirmed the reaction between the epoxide groups of the epoxy resin with the COOH groups of PLA and OH groups of the CP fiber. These reactions enhanced the interfacial adhesion between the PLA and CP fiber surfaces, which improved the mechanical properties, interfacial tension, morphology, thermal properties, and toughness of the PLA/epoxy/CP composites. The addition of 1 wt.% epoxy resin was found to be optimal for maximizing crosslinking, resulting in superior mechanical properties, contact angle, thermal properties, and crystallinity; at higher epoxy contents (3–10 wt.%), its plasticizer effects were more pronounced, leading to inferior mechanical properties, surface tension, thermal properties, and crystallinity. On the other hand, the increased plasticizer effect of the 3–10 wt.% PLA/Epoxy/CP composites improved their folding durability. The morphology of the PLA/Epoxy/CP composites showed fine distribution of CP fibers at 1 wt.% epoxy owing to optimal reactivity whereas, at higher epoxy amounts, large aggregates of CP fibers formed as defects in the composites. The lowest MFI was achieved at 1 wt.% epoxy owing to the crosslinking effect, and increased with increasing epoxy content due to the dominance of the plasticizer effect. A 3D filament was fabricated from PLA/Epoxy1/CP material using a twin-screw extruder, showing a tensile strength of 64.6 MPa and elongation at beak of 9.8%. This 3D bio-based polymer composite filament can be used to prepare injection mold prototypes for packaging, engineering, and medical prototype applications. PLA/Epoxy/CP composites can be used for other fabrication processes, such as injection molding, electrospinning, blown molding, and compression molding to prepare packaging, tissue engineering, plastic bags, and sample sheets.

## Figures and Tables

**Table 1:** Band assignment of PLA, CP, and epoxy.

Functional Groups	Wave Number in the Literature	PLA	Cassava	Epoxy
O–H stretching	3600–3300	-	3447	-
C–H stretching	2931	2946	2821	3059
C=O stretching	1740	1740	-	-
C=C stretching	1510, 1609	-	-	1510, 1609
–CH_3_ bending	1450	1450	-	1445
C–C stretching	1175	1175	-	-
C–O stretching	1200–800	850	1060, 893	912

**Table 2:** Thermal properties of PLA, PLA/CP, and PLA/Epoxy/CP composites containing 0.5–10 wt.% epoxy.

Sample	T_g_ (°C)	T_c_ (°C)	T_m_ (°C)	∆H_c_ (J/g)	∆H_m_ (J/g)	%X
PLA	62.3	-	153.5	-	33.8	36.1
PLA90/CP10	61.1	-	156.0	-	28.7	30.7
PLA/Epoxy0.5/CP	61.3	107.3	155.8	19.5	25.5	6.4
PLA/Epoxy1/CP	61.1	-	154.0	-	26.5	28.3
PLA/Epoxy3/CP	59.2	113.5	151.2	19.1	25.1	6.4
PLA/Epoxy5/CP	58.6	116.0	150.2	17.3	18.3	1.1
PLA/Epoxy10/CP	53.7	107.0	149.3	12.1	19.4	7.8

## Data Availability

All data in the article are included in this published article.

## Abbreviations

PLA, poly(lactic acid); CP, cassava pulp; AM, additive manufacturing; DSC, differential scanning calorimetry; FTIR, Fourier transform infrared spectroscopy; SEM, scanning electron microscopy; MFI, melt flow index; FE, folding endurance.

## References

[1] Hassan M.M., Tucker N., Le Guen M.J. (2020). Thermal, mechanical and viscoelastic properties of citric acid-crosslinked starch/cellulose composite foams. Carbohydr. Polym..

[2] Malhotra M., Garg N., Chand P., Jakhete A. (2023). Bio-based bioplastics: Current and future developments. Valorization of Biomass to Bioproducts.

[3] Arjmandi R., Hassan A., Zakaria Z. (2017). Polylactic Acid Green Nanocomposites for Automotive Applications. Green Biocomposites.

[4] Haafiz M.K.M., Hassan A., Arjmandi R., Marliana M.M., Fazita M.R.N. (2016). Exploring the Potentials of Nanocellulose Whiskers Derived from Oil Palm Empty Fruit Bunch on the Development of Polylactid Acid Based Green Nanocomposites. Polym. Compos..

[5] Jonoobi M., Harun J., Mathew A.P., Oksman K. (2010). Mechanical properties of cellulose nanofiber (CNF) reinforced polylactic acid (PLA) prepared by twin screw extrusion. Compos. Sci. Technol..

[6] Skorokhoda V., Semeniuk I., Peretyatko T., Kochubei V., Ivanukh O., Melnyk Y., Stetsyshyn Y. (2025). Biodegradation of polyhydroxybutyrate, polylactide, and their Blends by microorganisms, including Antarctic Species: Insights from weight Loss, XRD, and thermal Studies. Polymers.

[7] Joseph T.M., Kallingal A., Suresh A.M., Mahapatra D.K., Hasanin M.S., Haponiuk J., Thomas S. (2023). 3D printing of polylactic acid: Recent advances and opportunities. Int. J. Adv. Manuf. Technol..

[8] Chen X., Chen G., Wang G., Zhu P., Gao C. (2019). Recent Progress on 3D-Printed Polylactic Acid and Its Applications in Bone Repair. Adv. Eng. Mater..

[9] Arefin A.M.E., Khatri N.R., Kulkarni N., Egan P.F. (2021). Polymer 3D Printing Review: Materials, Process, and Design Strategies for Medical Applications. Polymers.

[10] Watcharamongkol T., Khaopueak P., Seesuea C., Wechakorn K. (2024). Green hydrothermal synthesis of multifunctional carbon dots from cassava pulps for metal sensing, antioxidant, and mercury detoxification in plants. Carbon. Resour. Convers..

[11] Jullanun P., Yoksan R. (2020). Morphological characteristics and properties of TPS/PLA/cassava pulp biocomposites. Polym. Test..

[12] Mohanty A., Misra M., Drzal L.T. (2001). Surface modifications of natural fibers and performance of the resulting biocomposites: An overview. Compos. Interface..

[13] Hsissou R., Seghiri R., Benzekri Z., Hilali M., Rafik M., Elharfi A. (2021). Polymer composite materials: A comprehensive review. Compos. Struct..

[14] Vieira M.G.A., da Silva M.A., dos Santos L.O., Beppu M.M. (2011). Natural-based plasticizers and biopolymer films: A review. Eur. Polym. J..

[15] Zeeman R., Dijkstra P.J., Van Wachem P., Van Luyn M., Hendriks M., Cahalan P., Feijen J. (1999). Crosslinking and modification of dermal sheep collagen using 1,4-butanediol diglycidyl ether. J. Biomed. Mater. Res. Off. J. Soc. Biomater. Jpn. Soc. Biomater. Aust. Soc. Biomater. Korean Soc. Biomater..

[16] Giustiniani A., Ilyas M., Indei T., Gong J.P. (2023). Relaxation mechanisms in hydrogels with uniaxially oriented lamellar bilayers. Polymer.

[17] (2013). Standard Test Method for Melt Flow Rates of Thermoplastics by Extrusion Plastometer.

[18] Panyathip R., Witthayapak M., Thuephloi P., Sukunta J., Thipchai P., Thanakkasaranee S., Jantanasakulwong K., Rachtanapun P. (2025). Characterization of corn husks carboxymethyl cellulose formation using Raman spectroscopy. Ind. Crops Prod..

[19] Wicaksono S., Chai G.B. (2012). A review of advances in fatigue and life prediction of fiber-reinforced composites. Proc. IMechE. Part L J. Mater. Des. Appl..

[20] Abdullah A.H.D., Chalimah S., Primadona I., Hanantyo M.H.G. (2018). Physical and chemical properties of corn, cassava, and potato starchs. IOP Conf. Ser. Earth Environ. Sci..

[21] Mandal A., Chakrabarty D. (2011). Isolation of nanocellulose from waste sugarcane bagasse (SCB) and its characterization. Carbohydr. Polym..

[22] Neto W.P.F., Silvério H.A., Dantas N.O., Pasquini D. (2013). Extraction and characterization of cellulose nanocrystals from agro-industrial residue–Soy hulls. Ind. Crops Prod..

[23] Mirhosseini H., Tan C.P., Aghlara A., Hamid N.S., Yusof S., Chern B.H. (2008). Influence of pectin and CMC on physical stability, turbidity loss rate, cloudiness and flavor release of orange beverage emulsion during storage. Carbohydr. Polym..

[24] Khatami M., Bairwan R.D., Khalil H.A., Surya I., Mawardi I., Ahmad A., Yahya E.B. (2024). The Role of Natural Fiber Reinforcement in Thermoplastic Elastomers Biocomposites. Fibers Polym..

[25] Thajai N., Rachtanapun P., Thanakkasaranee S., Punyodom W., Worajittiphon P., Phimolsiripol Y., Leksawasdi N., Ross S., Jantrawut P., Jantanasakulwong K. (2023). Reactive Blending of Modified Thermoplastic Starch Chlorhexidine Gluconate and Poly(butylene succinate) Blending with Epoxy Compatibilizer. Polymers.

[26] Xu J., Zhang J., Gao W., Liang H., Wang H., Li J. (2009). Preparation of chitosan/PLA blend micro/nanofibers by electrospinning. Mater. Lett..

[27] Acocella M.R., Corcione C.E., Giuri A., Maggio M., Maffezzoli A., Guerra G. (2016). Graphene oxide as a catalyst for ring opening reactions in amine crosslinking of epoxy resins. RSC Adv..

[28] Kiattipornpithak K., Thajai N., Kanthiya T., Rachtanapun P., Leksawasdi N., Phimolsiripol Y., Rohindra D., Ruksiriwanich W., Sommano S.R., Jantanasakulwong K. (2021). Reaction mechanism and mechanical property improvement of poly (lactic acid) reactive blending with epoxy resin. Polymers.

[29] Kanthiya T., Kiattipornpithak K., Thajai N., Phimolsiripol Y., Rachtanapun P., Thanakkasaranee S., Leksawasdi N., Tanadchangsaeng N., Sawangrat C., Wattanachai P. (2022). Modified poly (lactic acid) epoxy resin using chitosan for reactive blending with epoxidized natural rubber: Analysis of annealing time. Polymers.

[30] Yang S.-L., Wu Z.-H., Yang W., Yang M.-B. (2008). Thermal and mechanical properties of chemical crosslinked polylactide (PLA). Polym. Test..

[31] Rodríguez M.T., García S.J., Cabello R., Suay J.J., Gracenea J.J. (2005). Effect of plasticizer on the thermal, mechanical, and anticorrosion properties of an epoxy primer. J. Coat. Technol. Res..

[32] Laput O., Vasenina I., Salvadori M.C., Savkin K., Zuza D., Kurzina1 I. (2019). Low-temperature plasma treatment of polylactic acid and PLA/HA composite material. Polym. Biopolym..

[33] Wang P., Gao S., Chen X., Yang L., Wu X., Feng S., Hu X., Liu J., Xu P., Ding Y. (2022). Effect of hydroxyl and carboxyl-functionalized carbon nanotubes on phase morphology, mechanical and dielectric properties of poly (lactide)/poly (butylene adipate-co-terephthalate) composites. Int. J. Biol. Macromol..

[34] Ma C., Sánchez-Rodríguez D., Kamo T. (2021). A comprehensive study on the oxidative pyrolysis of epoxy resin from fiber/epoxy composites: Product characteristics and kinetics. J. Hazard. Mater..

[35] Bandyopadhyay A., Valavala P.K., Clancy T.C., Wise K.E., Odegard G.M. (2011). Molecular modeling of crosslinked epoxy polymers: The effect of crosslink density on thermomechanical properties. Polymer.

[36] Kiattipornpithak K., Rachtanapun P., Thanakkasaranee S., Jantrawut P., Ruksiriwanich W., Sommano S.R., Leksawasdi N., Kittikorn T., Jantanasakulwong K. (2023). Bamboo Pulp Toughening Poly(Lactic Acid) Composite Using Reactive Epoxy Resin. Polymers.

